# Metastatic extramammary Paget’s disease successfully treated by trastuzumab deruxtecan: a rare case report

**DOI:** 10.3389/fmed.2026.1831490

**Published:** 2026-06-24

**Authors:** Lien-Ping Chou, Ming-Yang Lee, Chien-Liang Fang, Chun-Liang Tung, I-Li Lin, Cheng-Huang Shen

**Affiliations:** 1 Department of Urology, Ditmanson Medical Foundation Chiayi Christian Hospital, Chiayi, Taiwan; 2 Department of Hematology and Oncology, Ditmanson Medical Foundation Chiayi Christian Hospital, Chiayi, Taiwan; 3 Division of Plastic and Reconstruction Surgery, Department of Surgery, Ditmanson Medical Foundation Chiayi Christian Hospital, Chiayi, Taiwan; 4 Department of Pathology, Ditmanson Medical Foundation Chiayi Christian Hospital, Chiayi, Taiwan; 5 Department of Radiology, Ditmanson Medical Foundation Chiayi Christian Hospital, Chiayi, Taiwan

**Keywords:** extramammory Paget disease, Next-generation sequencing, Paget disease, target therapy, trastuzumab deruxtecan

## Abstract

**Background:**

Metastatic extramammary Paget’s disease (mEMPD) is a rare cancer with a poor prognosis. Approximately 40% of cases overexpress the Her2/neu protein, making it a critical therapeutic target for precision-based systemic treatment.

**Case presentation:**

A 73-year-old man presented with a chronic erythematous patch on the left supra-pubic and scrotal area that had been slowly enlarging over months. A tender right groin mass developed recently. Imaging revealed right inguinal lymphadenopathy with necrosis, para-aortic lymph node involvement, bladder wall lesions, and right hydronephrosis. A skin biopsy confirmed the diagnosis of extramammary Paget’s disease (EMPD), and invasive EMPD was subsequently confirmed through the excision of metastatic lymph nodes in the right groin. The immunochemical stains of the carcinoma reveal low Her2/neu expression (++/+++). Next-generation sequencing (NGS) demonstrated a high tumor mutation burden (30Muts/Mb), along with ERBBR2, PIK3CA, and PTEN mutations. Initially, he received trastuzumab and pembrolizumab. However, there was no clinical response. Then, he was treated with trastuzumab deruxtecan (anti-Her2/neu antibody–drug conjugates, ADC). A partial response was noted clinically and radiologically, including the regression of lymphadenopathy and bladder lesion. EMPD with low Her2/neu expression and high TMB may benefit more from anti-Her2/neu ADC rather than from immune checkpoint inhibitors combined with anti-Her2/neu antibodies.

**Discussion:**

HER2-targeted strategies, including trastuzumab-docetaxel and T-DM1, show high efficacy in mEMPD. This paradigm shift toward molecularly driven, customized treatments optimizes clinical outcomes while maintaining patient performance status in this rare malignancy.

## Introduction

Metastatic extramammary Paget’s disease (mEMPD) is a rare and aggressive malignant disease that primarily affects the genital and axillary regions in elderly patients. While localized disease may be managed surgically, the prognosis for patients with lymph node or visceral metastases remains poor, with a 5-year overall survival (OS) rate of approximately 16% (1). Historically, the lack of prospective clinical trials has prevented the establishment of a standard consensus for systemic therapy. Conventional cytotoxic regimens, such as single-agent taxanes or platinum-based combinations, have been utilized; however, their efficacy is supported only by limited data. Treatment for mEMPD is thus guided by small case series involving chemotherapy with docetaxel, cisplatin, or 5-FU. The progression-free survival is approximately 5 to 7 months, and the overall survival is approximately 16 to 27 months (1).

The shift toward precision oncology has fundamentally altered this landscape, moving treatment from a purely histological approach to one that focuses on the molecular characteristics of the tumor. Research indicates that the immunohistochemical profile of mEMPD closely resembles that of breast cancer, with human epidermal growth factor receptor 2 (Her2/neu) overexpression or gene amplification observed in approximately 40% of cases. Consequently, Her2/neu has emerged as a promising target for the treatment of this advanced disease (17). Cases exhibiting Her2/neu overexpression can be treated with trastuzumab, and progression-free survival may be prolonged to 12 months (1). These findings underscore a paradigm shift toward molecularly targeted strategies that offer potent clinical activity with manageable safety profiles in this challenging malignancy. However, the effects of immunotherapy in EMPD are still undetermined; while some cases reports showed dramatic improvement, other studies have indicated only 2 months of progression-free survival.

## Case presentation

**Figure 1:**
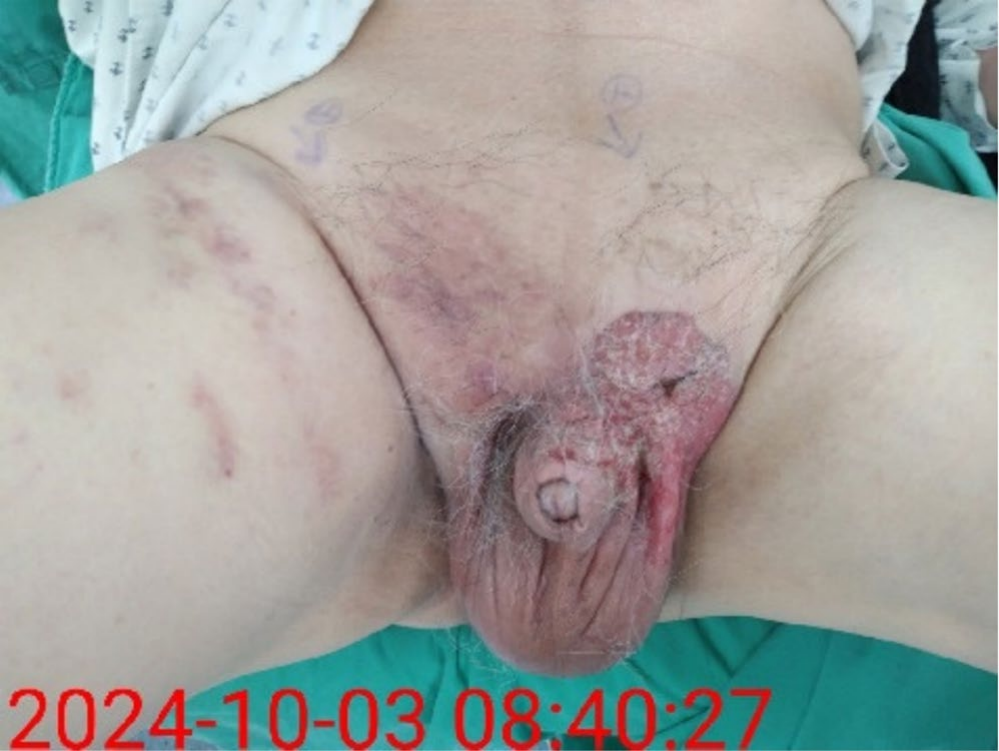
Images above demonstrate the patient’s preoperative inguinal ulcerative lesion.

**Figure 2:**
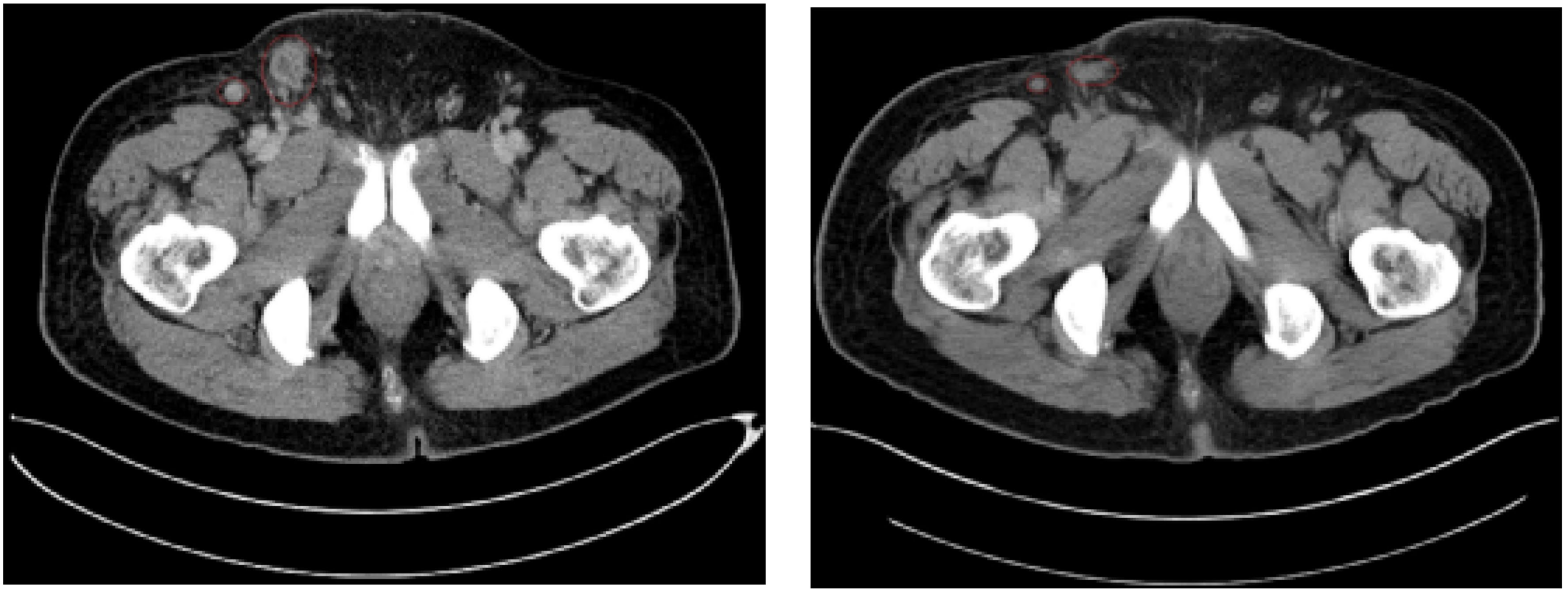
**(A,B)** Images above present a comparative view of the patient’s right inguinal lymph node in October 2024 and August 2025 following treatment with Herceptin. A marked reduction in the size of the right inguinal lymph node is observed after therapy.

A 73-year-old man with no significant history of any systemic diseases presented with one ill-defined erythema skin patch over the left suprapubic and left scrotum for years; this erythematous lesion began to extend slowly in recent months. A tender mass in the right groin (Figure 1) was noticed in the past week. There are no related symptoms such as pain, numbness in the limbs near the tumor, fluctuations in blood flow, or throbbing arteries. The computed venography revealed multiple enlarged lymph nodes in the right inguinal region (Figure 2), including one with central necrosis and an irregular, indistinct margin and some lymph nodes in the right pelvic sidewall and para-aortic space. On the other hand, two enhancing submucosal lesions were observed on the right lateral urinary bladder wall, along with right hydronephrosis and hydroureter extending to the right mid-segment of the ureter.

He first visited our dermatology outpatient clinic for a skin lesion biopsy on 25 September 2024, and the pathology report showed extramammary Paget’s disease. He was then transferred to our plastic surgery outpatient department, where a physical examination revealed one ill-defined erythematous patch measuring approximately 11×9 cm, with less discharge from the left suprapubic and left scrotum, a palpable and firm lymph node over the right groin region, and one ill-defined, black skin tumor measuring 6×5 mm on the glabella.

**Figure 3:**
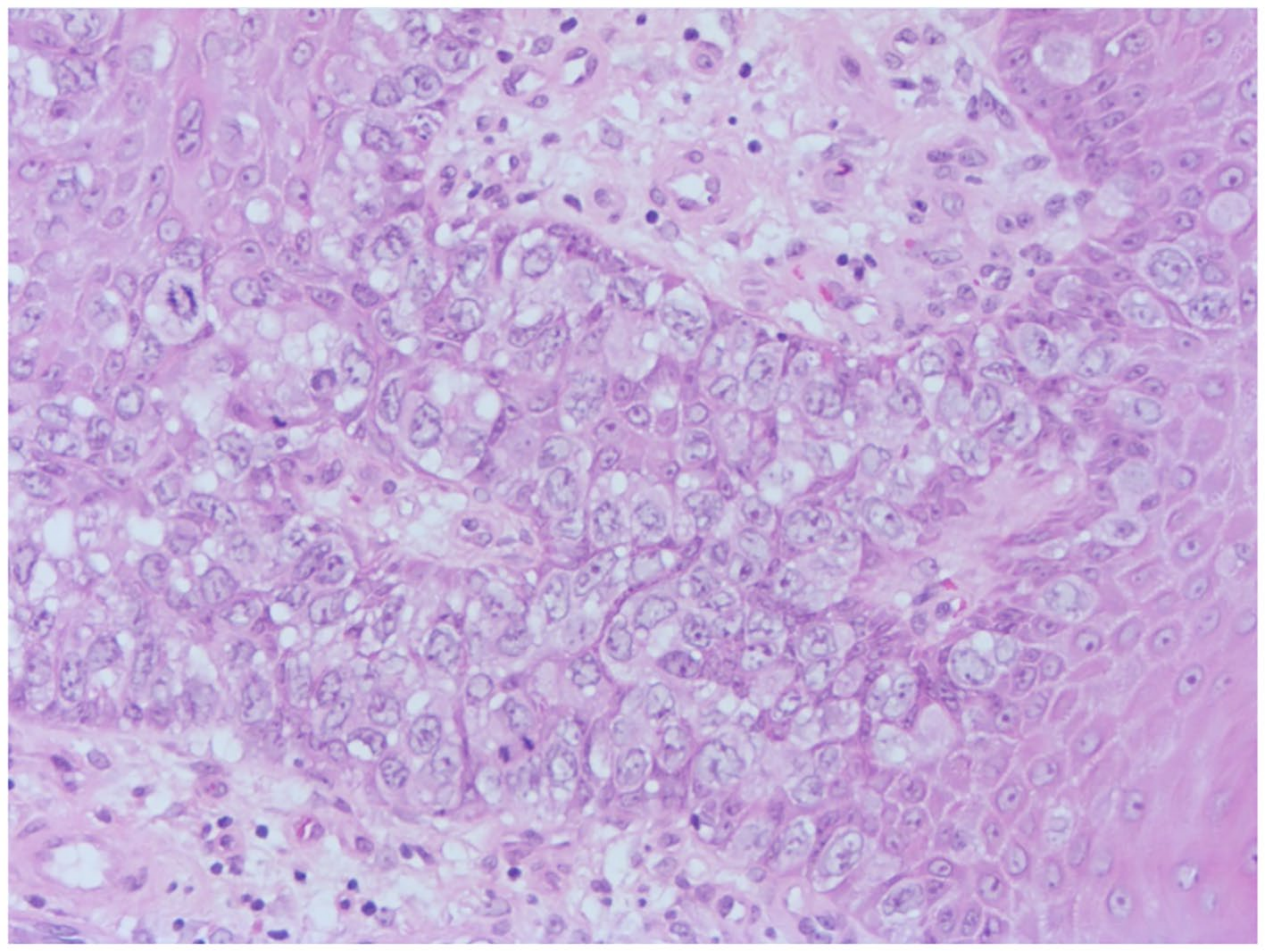
High magnification image shows reveal large, clear to pale Paget cells with abundant eosinophilic cytoplasm and moderate pleomorphism within the epidermis.

On 3 October 2024, a wide excision was performed using local advanced flap and full-thickness skin graft to treat Paget’s disease in the left suprapubic and left scrotum. Additionally, lymph nodes in the right groin region were excised, as well as a skin tumor on the glabella (Figure 1). The pathology report demonstrated extramammary Paget’s disease (EMPD) with focal invasion in the suprapubic and scrotum specimen (Figure 3). The report for the right groin lymph node also revealed that metastatic carcinoma with its immunochemical stains features [CK7(+), CK20(−), E-cadherin(+), GATA-3(+), TRPS-1(+), p63(−), NKX3.1(−), CDX-2(−). PSMA(−), polyclonalCEA(+), uroplakin(−), PAX8(−), and TTF-1(−). Her2/neu: Equivocal (++/+++)] was compatible with the diagnosis of EMPD (The primary site is located in scrotal and suprapubic areas. Typically, primary EMPD typically presents as CK7+/CK20 − and may be positive for GCDFP-15, whereas secondary EMPD of urothelial origin is frequently CK7+/CK20 + and positive for urothelial markers such as uroplakin II or III).

The immunochemical stains of the carcinoma reveal low Her2/neu expression (++/+++). Next-generation sequencing (NGS) demonstrated a high tumor mutation burden (30Muts/Mb) with ERBBR2, PIK3CA, and PTEN mutations (9–15).

Following the initial surgical intervention, the patient was commenced on targeted therapy. He initially received one cycle of trastuzumab, followed by four cycles of pembrolizumab monotherapy (200 mg) every 3 weeks. A restaging magnetic resonance imaging (MRI) scan performed in January 2025 showed stable disease; however, there was a lack of a significant clinical response. On the other hand, thickening of the right lateral wall of the urinary bladder was noted.

Based on the need for a more robust therapeutic approach, the treatment regimen was intensified in January 2025 to a combination of pembrolizumab (200 mg) and trastuzumab (330 mg) administered every 3 weeks. The transurethral resection of bladder tumor was performed in early April 2025. The pathological report revealed a poorly differentiated carcinoma that exhibited similar morphology to previous specimens (positive for CK7 (diffuse), GATA3 (diffuse) and NKX3.1 (focal) stains while negative for CK20, SATB2, CDX2, p63, uroplakin, INSM1 and synaptophysin stains), which supported a diagnosis of primary mEMPD with secondary bladder metastasis. A subsequent restaging MRI in April 2025 demonstrated a partial response, with a visible reduction in the size of the bilateral inguinal and paraaortic lymphadenopathy, as well as a decrease in the tumor within the bladder. However, despite the improvements in the internal radiological findings, the patient developed new skin lesions, which suggested cutaneous progression of the disease.

In response to the newly emerging skin lesions, the treatment was switched to trastuzumab deruxtecan (Enhertu), administered at a dose of 300 mg every 3 weeks. This antibody–drug conjugate specifically targets Her2/neu-positive cells. A restaging computed tomography (CT) scan of the chest and abdomen in August 2025 confirmed a continued response, showing a further decrease in the volume of the multi-site lymphadenopathy and the bladder tumor (Figure 2B).

The patient has tolerated the current regimen well, maintaining a good performance status. Throughout the course of these various systemic therapies, he has experienced no major adverse events, with mild fatigue being the only reported side effect. He continues to follow the current treatment regimen with ongoing clinical and radiological monitoring.

### Treatment

**Figure 4:**
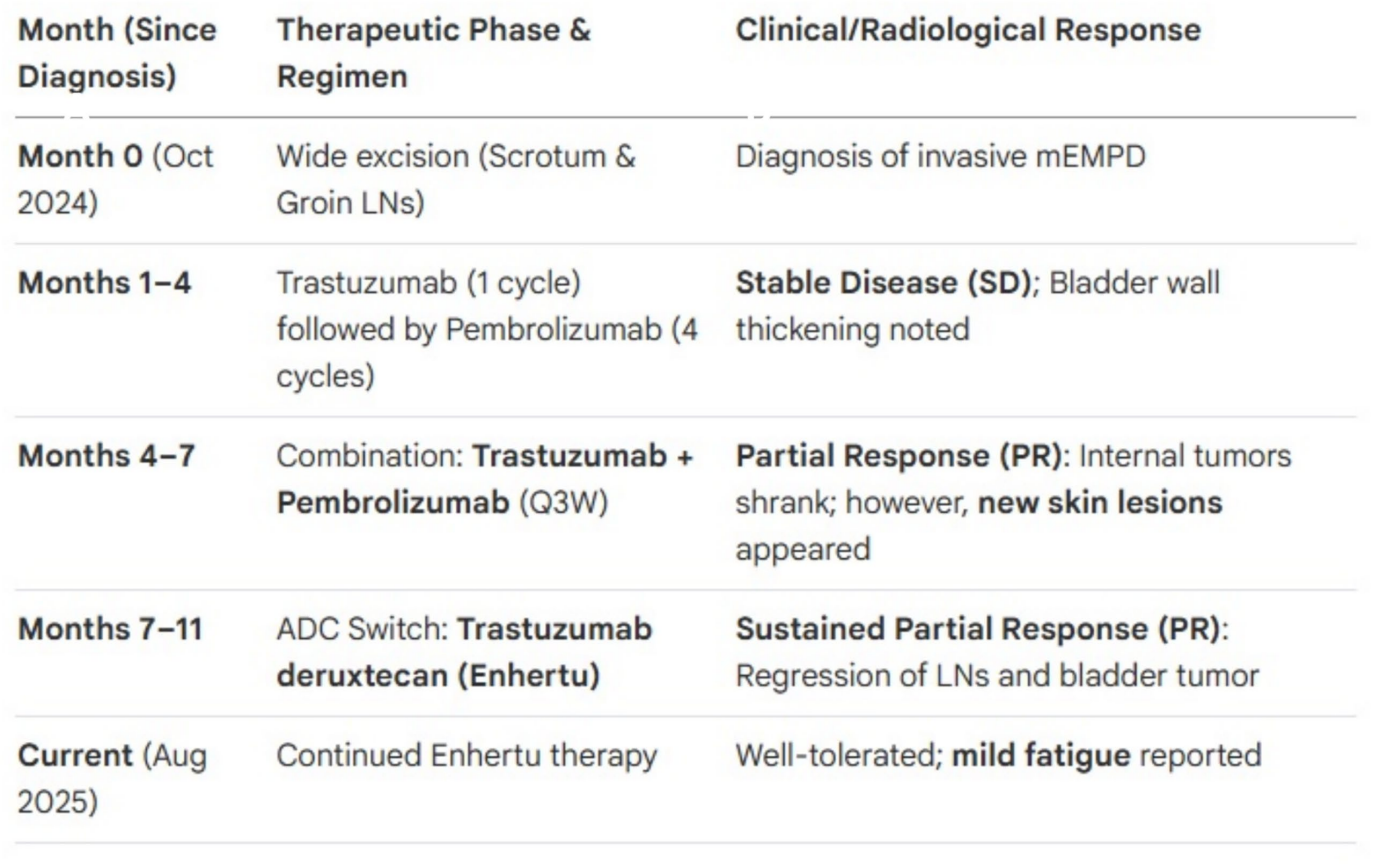
Treatment timeline has been organized into the table above, providing a comprehensive overview of the entire course from initial diagnosis to subsequent surgeries and pharmacologic therapies, including the number and timing of each treatment modality.

The patient was initially commenced on targeted therapy with one cycle of trastuzumab, followed by four cycles of pembrolizumab(200 mg) monotherapy. In January 2025, a restaging MRI showed stable disease with no significant clinical response. Consequently, the therapeutic regimen was modified to a combination of pembrolizumab (200 mg) and trastuzumab (330 mg), administered every 3 weeks. A subsequent restaging MRI in April 2025 showed a partial response, with a visible reduction in the size of the bilateral inguinal and paraaortic lymphadenopathy, as well as a decrease in the tumor within the bladder. However, due to the emergence of new skin lesions, the treatment was transitioned to trastuzumab deruxtecan (Enhertu 300 mg). The treatment timeline is summarized as a schematic illustration and presented in the figure at the end of the study (Figure 4).

### Outcome and follow-up

Following the initiation of trastuzumab deruxtecan, a restaging CT scan of the chest and abdomen in August 2025 confirmed a continued response, showing a further decrease in the volume of the multi-site lymphadenopathy and the bladder tumor. The patient continues to follow the current regimen, with restaging scans performed periodically to monitor for any signs of disease progression. In line with the management of other advanced malignancies that respond to targeted immunotherapy, the current clinical consensus is to continue treatment until there is disease progression or unacceptable side effects.

## Discussion

Metastatic extramammary Paget’s disease (mEMPD) is a rare and aggressive adeno-carcinoma that primarily affects older individuals, often localized in areas such as the skin of the genital or axillary area. The clinical prognosis for patients with distant metastases is poor, with 5-year survival rates estimated at only 16% (2). Due to its rarity, there is no universally accepted standard for systemic therapy. While traditional chemotherapy combinations, such as cisplatin, epirubicin, and taxanes, have been used, they typically offer only short-term benefits with significant toxicity.

The evolution of precision medicine has revealed that mEMPD often shares molecular characteristics with breast cancer, particularly the over-expression of the human epidermal growth factor receptor 2 (HER2), which is found in approximately 40% of patients (3). This discovery has made HER2-targeted therapy a cornerstone of modern treatment. Prospective data from the EMPD-HER2DOC phase II trial demonstrated that combining trastuzumab with docetaxel is highly effective, yielding an objective response rate (ORR) of 76.9% and a disease control rate of 100% (4). Furthermore, the antibody–drug conjugate trastuzumab emtansine (T-DM1) has shown even more significant activity in the TEMENOS-2 trial, achieving an investigator-assessed ORR of 85.7% in HER2-positive cases (5).

The clinical significance of this case lies in the patient’s HER2-low status (IHC 2 + with gene amplification). While approximately 40% of mEMPD cases overexpress HER2, the degree of expression often shows significant heterogeneity between the primary site and various metastatic deposits (6).

The success of trastuzumab deruxtecan (Enhertu) in this patient, despite the failure of standard trastuzumab combinations, can be attributed to the unique mechanism of next-generation ADCs (1, 17). Unlike first-generation ADCs (such as T-DM1 evaluated in the TEMENOS-2 trial), which require high receptor density, Enhertu utilizes a highly potent topoisomerase I inhibitor payload and a cleavable linker. This allows the payload to permeate the cell membrane and enter the neighboring tumor cells regardless of their individual HER2 expression levels, a phenomenon known as the bystander effect (1, 7). This mechanism is crucial in mEMPD to overcome Her2/neu heterogeneity, effectively eliminating mixed populations of HER2-high and Her2/neu-low cells within the tumor microenvironment (7).

A striking observation in this case was the complete lack of response to pembrolizumab despite a remarkably high tumor mutational burden (TMB 30 muts/Mb). While a high TMB is typically a robust predictor of response to immune checkpoint inhibitors (ICIs) in a tumor-agnostic setting (16), it appears insufficient in the context of EMPD.

Several mechanisms may underlie this resistance. The tumor microenvironment (TME) of EMPD is often “immunocompromised,” characterized by heavy infiltration of CD163 + M2 macrophages and FoxP3 + regulatory T cells (Tregs) (1). These cells suppress effector T-cell activation and create a biochemical barrier that facilitates immune evasion. This suggests that in rare malignancies such as EMPD, TMB is a limited predictive biomarker if the TME actively induces a highly suppressive immune state.

Recent systematic evidence confirms the evolving therapeutic landscape for HER2-positive disease. Incognito et al. (8) reported that sequential trastuzumab-containing treatments provide substantial clinical benefit, with an overall response rate (CR and PR) of 85.7% in metastatic vulvar Paget disease. Furthermore, prospective phase II evidence from the EMPD-HER2DOC trial demonstrated an ORR of 76.9% for a combination of trastuzumab and docetaxel (4), while the TEMENOS-2 trial showed that T-DM1 achieved an ORR of 85.7% in Japanese patients (5). This case extends these findings by demonstrating the viability of next-generation ADCs as a potent salvage therapy for patients who are refractory to standard anti-Her2/neu and immunotherapy regimens.

Alternative strategies, such as androgen blockade for tumors expressing androgen receptors or immune checkpoint inhibitors, are also being explored. Although typical markers such as PD-L1 expression are often absent in EMPD, rare cases of durable responses to immunotherapy suggest that a subset of patients may still benefit from these approaches. Overall, the management of mEMPD is shifting toward customized strategies that prioritize molecularly targeted agents, either as monotherapies or in combination with taxanes, to improve clinical outcomes while maintaining patient performance status.

## Data Availability

The original contributions presented in this study are included in the article and/or supplementary material. Further inquiries can be directed to the corresponding author.
